# Subthalamic nucleus deep brain stimulation and impulsivity in Parkinson’s disease: a descriptive review

**DOI:** 10.1007/s13760-021-01684-4

**Published:** 2021-05-07

**Authors:** Viviana Lo Buono, Marietta Lucà Trombetta, Rosanna Palmeri, Lilla Bonanno, Emanuele Cartella, Giuseppe Di Lorenzo, Placido Bramanti, Silvia Marino, Francesco Corallo

**Affiliations:** IRCCS Centro Neurolesi Bonino Pulejo, Messina, Italy

**Keywords:** Impulsivity, Impulse control disorders, Deep brain stimulation, Decision-making, Parkinson’s disease

## Abstract

Standard treatment of Parkinson’s disease involves the dopaminergic medications. Deep brain stimulation of the subthalamic nucleus (STN-DBS) is an important neurosurgical intervention often used as alternative treatment to drug therapy; however, it can be associated with increase of impulsive behaviors. This descriptive review focused on studies investigating the correlation between Deep brain stimulation of the subthalamic nucleus and impulsivity in Parkinson’s disease patients, arguing, the action’s mechanism and the specific role of the subthalamic nucleus. We searched on PubMed and Web of Science databases and screening references of included studies and review articles for additional citations. From initial 106 studies, only 15 met the search criteria. Parkinson’s Disease patients with and without Deep Brain Stimulation were compared with healthy controls, through 16 different tasks that assessed some aspects of impulsivity. Both Deep brain stimulation of the subthalamic nucleus and medication were associated with impulsive behavior and influenced decision-making processes. Moreover, findings demonstrated that: Impulse Control Disorders (ICDs) occurred soon after surgery, while, in pharmacological treatment, they appeared mainly after the initiation of treatment or the increase in dosage, especially with dopamine agonists. The subthalamic nucleus plays a part in the fronto-striato-thalamic-cortical loops mediating motor, cognitive, and emotional functions: this could explain the role of the Deep Brain Stimulation in behavior modulation in Parkinson’s Disease patients. Indeed, increase impulsivity has been reported also after deep brain stimulation of the subthalamic nucleus independently by dopaminergic medication status.

## Impact and implications

Deep brain stimulation of the sub-thalamic nucleus (STN-DBS) is an important neurosurgical intervention that could determine, as well as dopaminergic medication, relevant side effects such as increased impulsivity.

The studies analyzed in this review showed that after the surgical intervention, impulsivity improves independently by dopamination medication status. These findings could help to individuate contrasting data about the role of DBS on impulsivity in PD patients.

Future research should include the study of other factors, such as genetic predisposing, direct effect on limbic part of STN, cognitive outcome or depression scores, and should conduct larger, prospective, controlled trials to better clarify how different subcomponents of impulsivity can be modulated both by dopaminergic drugs and STN-DBS.

## Introduction

Parkinson’s disease (PD) is a neurodegenerative disorder due to loss of dopaminergic neurons in the substantia nigra pars compacta (SNc) and it is characterized by tremor, rigidity, and bradykinesia [1]. Although classically defined by motor symptoms, it is also associated with non-motor manifestations [2] that have a negative impact on the quality of life of patients [3]. Non-motor symptoms include autonomic and sensory dysfunctions, such as pain and a loss of smell or hyposmia, sleep disorders, cognitive and mood alterations [4–7]. Standard treatment for PD involves dopamine precursor of levodopa and dopamine agonists (DA) [8]. However, drug therapy is associated with side effects: on–off phenomena due to pharmacokinetics, levodopa-induced dyskinesia (LID), or non-motor symptoms including impulse control disorders (ICDs), such as gambling, hypersexuality, overeating [9]. Particularly, impulsive behaviors are increasingly reported as serious side effects of dopaminergic medication, used in the treatment of PD. Indeed, in the course of dopaminergic treatment patients can show impulsivity characterized by an inability to resist an inappropriate behavior. DA therapy is a major risk factor for the development of ICDs [10]. Impulsivity is characterized by a tendency towards rapid, ill considered, disinhibited choices. It can be broadly divided into:

- decisional forms, including delay discounting (preference of a small immediate over a larger delayed reward);
- reduced sensitivity to adverse outcomes (negative prediction errors) during learning;
- reflection impulsivity (rapid decision-making);
- risk-taking and response conflict (slowing and errors with competing responses);
- motor forms, such as response inhibition (inhibition of a response whereby individuals are biased to make a specific response because it is repeated or more frequent) [11].

Deep brain stimulation (DBS) is an adjunctive therapy to reduce some of the symptoms of an advanced stage that responds to levodopa Deep brain stimulation (DBS) is an adjunctive therapy in reducing some of the symptoms of advanced, levodopa-responsive. It improves motor disability by 33%—67%, motor fluctuations by 73%—83%, and dyskinesias caused by levodopa [7, 13–15]. DBS uses electrodes connected to a device called ‘implantable pulse generator” that delivers electrical stimuli to a specific brain region. The subthalamic nucleus (STN) is a cerebral area commonly target for DBS in PD [16]. STN has been regarded as a significant structure in the modulation of the activity of output basal ganglia structures; it has an essential role in motor functions, but it has been linked both to reward and to inhibitory control. Bilateral continuous high-frequency stimulation of the STN with typically high frequency (130 to 185 Hz), with pulse widths’ amplitudes of 60 to 120 μsec at voltages ranging from 2.0 to 5.0 V, has been used to treat PD, although it is sometimes related to side effects that could worsen the quality of life, how as reported by Funkiewiez et al. [17]. This study found an increase of cognitive and neuropsychiatric symptoms after STN-DBS, such as impairment in verbal fluency and executive functions, to lack motivation, depression, mania, apathy and explosive-aggressive behavior. In addition, also STN-DBS, as well as drug therapy, has been associated with an increased risk of ICDs [18].

This descriptive review focuses on the studies that investigated the effect of STN-DBS on impulsivity in PD patients and argued its possible mechanisms of action.

## Material and methods

### Search strategy

Studies were identified in PubMed (2003, year of the first related published article — January 2020) and Web of Science databases (November 2007 — January 2020). The search combined the following terms: ("impulsive behavior" [MeSH Terms] OR ("impulsive" [All Fields] AND "behavior" [All Fields]) OR "impulsive behavior" [All Fields] OR "impulsive" [All Fields]) AND ("deep brain stimulation" [MeSH Terms] OR ("deep" [All Fields] AND "brain" [All Fields] AND "stimulation" [All Fields]) OR "deep brain stimulation" [All Fields]) AND ("parkinson disease" [MeSH Terms] OR ("parkinson" [All Fields] AND "disease" [All Fields]) OR "parkinson disease" [All Fields] OR "parkinsons" [All Fields]). The search terms were identified in the title and abstract. We selected only English texts. After duplicates had been removed, articles were evaluated based on the title, abstract, and text. Studies that examined impulsivity in PD patients were included, after they fulfilled the following criteria:

1. The sample population included PD patients with STN DBS;
2. Studies provided a neuropsychological assessment of impulsivity and neurocognitive performances;
3. Data compared the performance of PD patients on/off stimulation and on/off medication and healthy controls (HC);
4. We excluded studies on PD patient with dementia or affected by other neurological or major psychiatric disorders;
5. Animal studies and published in non-peer reviewed research were excluded;
6. We excluded case studies.

**Fig. 1:**
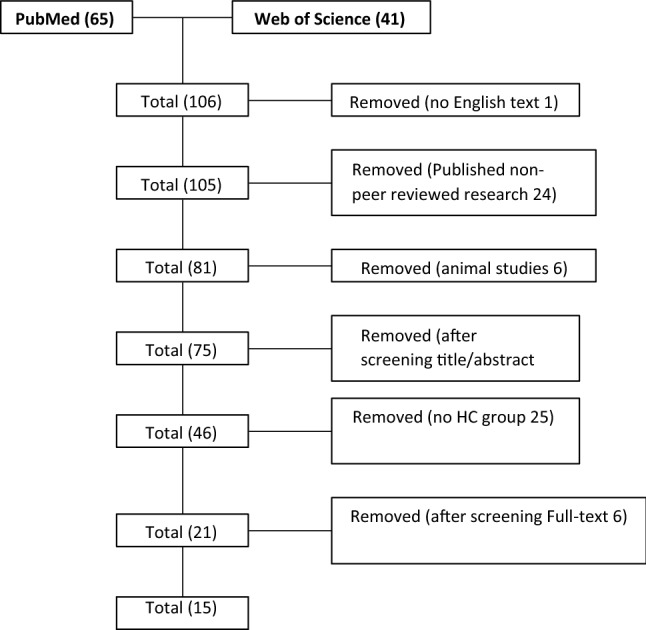
Search and selection of eligible article

Of 108 studies identified, 15 met the inclusion criteria (Fig. 1). All studies conducted research on PD patients divided into two groups: PD with STN-DBS (a subgroup treated with both L-dopa and DA, ones treated only with L-dopa, and the last without medication treatment) and PD without DBS (three subgroups: PD NO-DBS treated with both L-dopa and DA, PD NO-DBS treated only with L-dopa, and PD NO-DBS without medication treatment). All PD patients fulfilled diagnostic criteria for PD according to the United Kingdom Parkinson Disease Society Brain Bank for Idiopathic PD [19]. Disease severity was rated on the Unified Parkinson Disease Rating Scale motor score and the Hoehn and Yahr score (stages I–III) [20].

The HC groups consisted of volunteers without a history of neurological or psychiatric disorders. All subjects, matched for age and education, had normal or corrected to normal vision.

In these studies, impulsivity was evaluated by 16 different tasks (Table 1). Demographic characteristics of each study’s sample were shown in Table 2.

**Table 1:** Studies assessing impulsivity and neuropsychological functions in PD patients with and without STN DBS

Authors (year)	Aim	Sample (*n*)	Task to evaluate impulsivity	NPS evaluation	Results
Frank et al. (2007)	To test two distinct mechanisms by which DBS and dopaminergic medication can cause impulsive behavior	17 PD STN DBS + L-dopa 15 PD L- Dopa 27 HC	Probabilistic selection task	MMSE NAART Hoehn- Yahr Scale	PD STN-DBS more impulsive than PD L- DOPA and HC
Coulthard et al. (2012)	To investigate the effect of both dopamine and STN- DBS on behaviour by testing PD patients with dopamine replacement therapy and/or STN DBS	11 PD STN DBS + L-dopa 11 PD L- dopa 15 HC	Decision-making task	MMSE DRS2 AEMSS LARS BIS UPDRS	PD STN-DBS more impulsive than PD L- DOPA and HC
Djamshidian et al. (2014)	To assess perceptual decision-making in PD patients with bilateral STN-DBS	14 PD STN DBS + L-dopa + DA 13 PD STN-DBS + L-dopa 17 HC	Perceptual inference task	MMSE UPDRS	PD STN-DBS L-dopa + DA more impulsive than PD STN-DBS L-dopa and HC
Pote et al. (2016)	To investigate whether combined effects of STN stimulation and under pressure condition lead to greater impulsivity in PD	12 PD STN DBS + L-dopa 12 HC	Moving-dots task	MMSE BDI SAS Hoehn- Yahr Scale	PD STN DBS more impulsive than HC
Brandt et al. (2015)	To determine whether DBS of the STN is associated with faulthy decision- making due to increased risk-taking among PD patients	15 PD STN DBS + L-dopa 15 PD L- dopa 15 HC	Game of Dice Task. Deal or No-Deal Framing Paradigm	MoCA HART GDS-15 QUIP Hoehn- Yahr Scale UPDRS	PD STN DBS more impulsive than PD Non-DBS/HC on risky decision-making situation PD Non-DBS/HC more impulsive than PD STN DBS on ambiguous risk situation (PD DBS more conservative)
Boller et al. (2014)	To investigate the influence of dopaminergic medication and STN- DBS on decision-making under risk	18 PD STN DBS + L-dopa 18 HC	GDT	MDRS BDI PANDA MMSE Verbal Fluency	A positive influence of both MED and STN-DBS on making decisions under risk
Mirabella et al. (2012)	To assess the role of the right STN in the inhibitory control	10 PD STN DBS + L-dopa + DA	Countermanding reaching task Go-only task	Hoen-Yahr Scale UPDRS	Inhibitory control is improved only when both DBS are active
		13 HC			Bilateral stimulation of STN restores the inhibitory control to a near-normal level
Georgiev et al. (2016)	To explore the effect of STN-DBS on the probabilistic Go/NoGo task in PD	20 PD STN DBS + L-dopa + DA 10 PD L- dopa + DA	Go/NoGo task	UPDRS	Movement Time: PD STN-DBS more impulsive than PD and HC Reaction Time: PD STN-DBS and PD < HC
Aiello et al. (2017)	To assess whether an increased level of motivation for food reward and/or a deficit in response control may be responsible for weight gain after STN-DBS in PD patients	18 PD (evaluated before and after STN DBS) + L-dopa 18 HC	Food Go/NoGo task	MMSE BDI SHAPS- anhedonia BIS UPDRS Test Battery to assess food reward sensitivity	PD after STN-DBS more impulsive than PD before STN-DBS/HC Positive correlation between weight gain and wanting for low calories foods. No association with food Liking PD after STN-DBS more impulsive than PD before STN- DBS/HC Positive correlation between weight gain and attentional impulsiveness
Wylie et al. (2010)	To investigate the effect of STN DBS on the ability to suppress incorrect response impulses to facilitate the selection of goal-directed actions in PD	8 PD STN DBS + L- dopa + DA 8 PD STN DBS + L- dopa 1 PD STN DBS 17 HC	Simon task	MMSE UPDRS	PD STN DBS more impulsive than HC
van Wouwe et al. (2017)	Experiment 1: to investigate the effect of chronic versus acute STN DBS on inhibitory control in PD patients OFF dopaminergic medication Experiment 2: To test whether stimulating STN subregions differentially modulate proactive response control and the proficiency of reactive inhibitory control	Exp 1: 12 PD STN DBS + L-dopa Exp 2. 11 PD with bilateral STN-DBS + L-dopa 22 HC	Simon task	MMSE CESD BDI UPDRS	Exp 1: PD STN DBS = HC DBS improves reactive inhibitory control Exp 2: PD ventral STN DBS more impulsive than PD dorsal STN DBS and HC Dorsal vs ventral STN is more crucial for modulating the reactive inhibitory control of motor actions
Florin et al. (2013)	To analyse pathological changes in risk-taking behaviour of PD patients with STN- DBS in a social context	30 PD STN DBS + L-dopa 29 PD + L-dopa 23 HC	Addition task in a social and competitive context	UPDRS III BDI BIS	PD STN-DBS more impulsive than PD Non-DBS/HC
Plessow et al. (2014)	To understand whether and how the basal ganglia are involved in the interplay between automatic and habitual response impulses and inhibitory control	13 PD STN DBS **(without L-dopa medication)** 26 HC	Spatial-compatibility task	UPDRS	DBSON = improvement of automatic response activation DBSOFF = reduction of impact of automatic response activation
Djamshidian et al. (2013)	To assess the role of dopamine agonist therapy and DBS on reflection impulsivity in PD patients	20 PD L- dopa + DA 14 PD L- dopa 16 PD STN DBS + L-dopa + DA 11 PD STN DBS + L-dopa 18 HC	Beads task	MMSE UPDRS	All PD treated with DA More impulsive than other PD groups and HC
Tessitore et al. (2017)	To investigate intrinsic neural networks connectivity changes in Parkinson's disease (PD) patients with and without impulse control disorders (ICD)	15 PD with ICD (ICD +), 15 PD without ICD (ICD −) (all treated with L-Dopa and DA) 24 HC	MIDI (compulsive buying, pathological gambling hypersexuality, compulsive eating and punding)	MMSE	The study confirmed the crucial role of an abnormal prefrontal-limbic-striatal homeostasis in their development

*BDI* Beck Depression Inventory, *BIS* Barratt Impulsiveness Scale, *CESD* Center for Epidemiologic Studies Depression Scale, *DRS2 AEMSS* age-adjusted score for the dementia-rating scale, *GDS* Geriatric Depression Scale, *GDT* Game of Dice Task, *HART* Hopkins Adult Reading Test, *HC* healthy control, *LARS* Lilly Apathy Rating Scale, *MDRS* Mattis Dementia Rating Scale, *MMSE* Mini-Mental State Examination, *MoCA* Montreal Cognitive Assessment, *NAART* North American Adult Reading Test, *PANDA* Parkinson Neuropsychometric Dementia Assessment, *PD* Parkinson’s disease, *STN-DBS* Deep Brain Stimulation of the subthalamic nucleus, *QUIP* Questionnaire for Impulsive/Compulsive Disorders in Parkinson’s Disease-short form, *SAS* Starkstein Apathy Scale, *SHAPS* Snaith-Hamilton Pleasure Scale, *UPDRS III* Unified Parkinson’s Disease Rating Scale Part III, *QUIP-RS*: Questionnaire for Impulsive-Compulsive disorders in PD Rating Scale

**Table 2:** Demographic characteristics of the sample of studies

Authors, Published (year)	Sample (participants)	Mean age (years)	Years of education	PD Disease duration (years)	DBS (years)
Frank et al. [21]	HC	66.0 ± 1.7	16.2 ± 0.7		
	PD STN DBSON	64.5 ± 2.8	14.2 ± 1.5	14.4 ± 1.5	
	PD STN DBSOFF	62.3 ± 3.3	14.4 ± 1.2	15.2 ± 1.8	
	PD MEDON	67.8 ± 2.1	17.8 ± 1.2	8.8 ± 0.8	
	PD MEDOFF	67.6 ± 2.5	19.2 ± 1.4	9.5 ± 1.4	
Coulthard et al. [22]	HC	57 ± 3.4			
	PD STN DBS	56 ± 2.3		9.5 ± 0.8	2.21
	PD MED	58.9 ± 2.0		7.27 ± 1.65	
Djamshidian et	HC	59.9 ± 10.4			
al. [23]	PD STN DBS + L-	60.0 ± 7.2		13.3 ± 4.8	3.6 ± 2.4
	dopa	55.9 ± 10.0		15.0 ± 4.9	3.9 ± 2.3
	PD STN DBS + L-				
	dopa + DA				
Pote et al. [24]	HC	60.67 ± 10.58	16.96 ± 3.63		
	PD STN DBS +	56.75 ± 5.36	14.50 ± 3.37	12.58 ± 3.55	2.58 ± 1.02
	L-dopa				
Brandt et al. [25]	HC	62.39 ± 10.04	16.00 ± 2.20		
	PD STN DBS	67.15 ± 6.28	16.00 ± 2.73		
	PD MED	64.78 ± 8.09	16.27 ± 2.96		
Boller et al. [26]	HC	63.7 ± 9.0			
	PD STN DBS	64.3 ± 10.2		13.61 ± 7.11	2.3 ± 2.6
Mirabella et al. [27]	HC	60.7 ± 1.3			
	PD STN DBS	60.1 ± 1.8		17.5 ± 1.6	3.5 ± 0.4
Georgiev et al. [28]	HC	54.00 ± 7.09	14.20 ± 2.74		
	PD STN DBS + L-	56.77 ± 8.93	13.18 ± 2.42	15.27 ± 4.34	3.30 ± 1.25
	dopa + DA	57.70 ± 7.76	13.50 ± 2.41	13.30 ± 5.54	
	PD L-dopa + DA				
Aiello et al. [29]	HC	61.6 ± 8.9	11.8 ± 2.7		
	PD STN DBS	60.2 ± 6.9	9.9 ± 4.6	9.8 ± 4.2	
Wylie et al. [30]	HC	62.6 ± 8.4	16.7 ± 3.1		
	PD STN DBS	61.8 ± 7.6	15.7 ± 3.2	13.8 ± 5.9	
Van Wouwe et al. [31]	HC PD STN DBS (Exp 1) PD STN DBS (Exp 2)	63.9 ± 1.6 59.3 ± 2.9 58.9 ± 2.6	14.5 ± 0.7 13.7 ± 0.6 13.7 ± 0.7	11.8 ± 1.8 12.9 ± 1.7	
Florin et al. [32]	HC PD STN DBS PD	56.5 ± 7.2 57.9 ± 9.4 57.4 ± 9.2		11.2 ± 6.5 5.3 ± 3.7	
Plessow et al. [33]	HC PD STN DBS	65.08 ± 8.14 64.31 ± 6.59	13.96 13.77	17.00 ± 6.03	1.69 ± 1.18
Djamshidian et al. [34]	HC	58.9 ± 12.8	13.6 ± 3.2		
	PD STN DBS + L-	59.1 ± 11.6	13.7 ± 2.8	15.6 ± 6.0	3.6 ± 2.2
	dopa + DA	57.0 ± 7.0	13.9 ± 2.8	14.4 ± 5.0	3.4 ± 3.3
	PD STN DBS + L-	64.3 ± 5.2	14.5 ± 2.5	11.1 ± 7.0	
	dopa	67.2 ± 7.5	14.8 ± 3.1	6.2 ± 3.8	
	PD L-dopa + DA				
	PD L-dopa				

*HC* health controls, *PD* Parkinson’s Disease Patients, *STN-DBS* deep brain stimulations of the subthalamic nucleus, *DA* dopamine agonist therapy

## Results

The role of DBS in ICD is not entirely clear and studies here identified have revealed conflicting results. Indeed, some authors affirmed that hyperdopaminergic manifestations, such as ICDs, could be reduced after DBS, in association with a reduction in the dose of levodopa [35]. These studies argued dopaminergic therapy, particularly DA, could cause the onset of ICDs, [36, 37]. Others authors, instead, reported that DBS is greater involved in increasing ICDs than L-dopa medication [21, 26].

### STN-DBS and dopamine replacement in decision-making and impulsive behavior

Many studies have evaluated decision-making process in PD, comparing patients with STN DBS and patients treated pharmacologically. Findings revealed that both STN-DBS and medication were associated with impulsive behavior and both of them influenced decision-making processes in risky situations [21, 25]. According to these data, Djamshidian et al. [36] suggested that DA rather than DBS was responsible for the inability to slow down in high-conflict situations. Moreover, DA combined with L-dopa and STN-DBS would be causing sensitization of mesolimbic dopamine levels resulting in reduced decision threshold in a perceptual decision-making task. However, in a subsequent study, Djamashidian (2014), using the Beads task, had reported contrasting results showing that neither STN-DBS nor L-dopa monotherapy increased impulsive choices in PD patients,. However, this result could be due to a methodological problem related to the low sensitivity of the test chosen to evaluate impulsive behavior. Indeed, in both studies (Djashidian et al. 2013, 2014), the author concluded that all patients treated with L-dopa in combination with DA were more impulsive than all other groups treated with DBS, confirming the hypothesis that it is the pharmacological therapy rather than DBS that causes more impulsivity tendency.

Reference [26] highlighted the role of STN-DBS in the choice on the basis of probabilistic information from multiple stimuli regardless of the ON or OFF pharmacological status. In this study, patients performed the tasks ON or OFF DBS and/or ON or OFF dopaminergic therapy. When dopaminergic therapy was in OFF, memory for probabilistic information was compromised; while in OFF STN-DBS, the decision-making in combined multiple pieces of information was interrupted. Findings demonstrated that both dopamine medication and STN-DBS could influence decision-making processes but acting on different levels within the same task.

### The STN-DBS effects on impulsive behavior

Studies about Decision-making studies in ambiguous or risky contexts in on PD patients with DBS, have shown that STN-DBS induces a lowering of the response threshold and a decrease in the level of caution by altering the speed-accuracy trade-off. In addition, stimulation increases rapid response errors and this effect is greater in BDS ON than in BDS OFF [24]. In addition, STN-DBS influences the pathways involved in risk assessment leading to a combination of overestimation of patient performance and increased risk-taking, and preference for competitive environments [23]. In a study assessing willingness to gamble on a fixed (unambiguous) prize, non-surgical PD patients tended to be more risk-averse than HC, whereas DBS patients were more willing to gamble for gains, as well as, losses both ON and OFF stimulation. On “risky” decision-making tasks, DBS patients were more risk-taking than normal, but stimulation might temper this tendency. Moreover, STN-DBS resulted in the performance of the patients to become differentially faster and more erroneous and induced a decrease in the level of caution [38].

Other studies on impulsivity in PD patients mainly focused on motor impulsivity using the stop-signal reaction time (RT) task [27, 28] and Go/NoGo RTs. Motor impulsivity results from insufficient motor or response inhibition. In PD patients, STN-DBS selectively improved inhibitory functions as its electrical stimulation significantly shortened the stop-signal reaction time [27]. Moreover, STN-DBS selectively decreased discriminability when the response was most prepotent [28]. Movement execution resulted faster with STN stimulation than with DBSOFF across different Go probability levels. Furthermore, these studies found that in comparison to HC, both STN-DBS and unoperated PD patients were more prone to making anticipatory errors.

Reference [29] examined more specifically the role of the subthalamic nucleus in reward and inhibitory control through go/no go tasks. The authors evaluated whether the weight gain experienced by PD patients after STN-DBS may be due to an alteration of reward and inhibitory functions. The results showed that body weight increased significantly after STN-DBS since to increased impulsivity and reward sensitivity. As impulsivity and reward sensitivity increased.

The effects of DBS on impulsivity were also investigated by other tasks involving inhibitory control or response selection under conflict. In Simon task, that produced conflict from response impulses in patients with PD and HC, responses were faster and more accurate when relevant (color) and irrelevant (spatial location) features of an imperative stimulus corresponded to the same response, but slower and less accurate when these features signaled conflicting responses [30]. STN-DBS patients were more susceptible to reacting impulsively in situations requiring a speedy decision among highly conflicting response alternatives. Moreover, STN-DBS patients overestimated their own performance assuming an extremely risky gambling behavior assessed by a simple two-choice gambling task due to the modulation of basal-ganglia-cortex circuits by STN-DBS leading to overly competitive behavior [23].

Since it is not yet clear what aspects of PD are actually caused by Basal Ganglia (BG) dysfunction, [34] investigated 13 patients with PD off-medication with bilateral subthalamic deep brain stimulation (DBS) with and without stimulation (DBS ON and DBS OFF, respectively) and 26 HC. All participants performed a task that verifies the relationship between the automatic response impulses and the selection of the direct action to the target. The results showed an improvement in automatic response activation under DBS ON, increasing susceptibility to impulsive responses, and a reduced impact of automatic response activation under DBS OFF. These data seem to support or argue that the BG determines the efficiency of the regulation and transmission of stimulus-driven bottom–up response activation necessary for efficient response selection.

### Neuroanatomical correlates of impulsive behavior

Stimulation of the ventromedial STN through its close connection to the nucleus accumbens loop potentially induces ICD [32]. These cerebral areas are crucial in impulse control, motivational processes, and addictive behaviors. In addition, the orbitofrontal and anterior cingulate cortices and the ventral striatum are linked to impaired risk evaluation, which is mediated by DA in PD patients suffering from comorbid ICD. In recent years, magnetic resonance imaging examinations (MRI) focused on the dopaminergic system, have significantly contributed to the knowledge of neurobiological factors for ICDs. In PD patients with ICDs, structural MRI revealed orbitofrontal atrophy [33]. In addition, connectivity dysfunction between the striatal and limbic areas involving the neurocognitive networks has been proposed. In particular, a decreased of cerebral connectivity was found in the central executive network (mediofrontal areas, anterior cingulate and paracingulate cortices), while increased connectivity has been identified in the salience network (limbic-paralimbic network) and in the default mode network (precuneus and posterior cingulate, bilateral and ventromedial cortices) [31].

STN plays a key role in inhibition processes, which permit the suppression of premature actions and to block interference from irrelevant stimuli. Altered decision-making is associated with cognitive impulsivity, which is considered the inability to weigh the consequences of immediate and future events and, consequently, delay gratification [39]. Lesion studies have suggested the ventromedial prefrontal cortex, is the main area involved in this type of impulsivity [40]. A study conducted by van Wouwe et al. [41] reported that STN-DBS ON stimulation improved the reactive inhibition of impulsive actions that interfere with goal-directed behavior. These findings showed that DBS improves reactive inhibitory control, regardless of medication and regardless of whether it concerns chronic or acute Subthalamic Nucleus stimulation. The most important result of this study was that especially the dorsal STN circuitries were crucial for modulating the reactive inhibitory control of motor actions, how the selective stimulation of dorsal and ventral subregions of the Subthalamic Nucleus had indicated.

### STN-DBS could improve ICD

Two studies hypothesized that STN DBS selectively improves inhibitory functions [27, 41]. This finding is consistent with the idea that DBS not only shuts off the pathological activity of STN but also imposes a new pattern of activity with beneficial effects. The improvement in the proficiency of inhibitory control would be greatest when stimulating a relatively dorsal STN subregion compared to a relatively ventral STN subregion. Confirmation of this pattern would provide new evidence that dorsal STN circuitries play, direct role in reactive inhibitory motor control processes.

## Discussion

Studies on impulsivity in PD patients highlighted conflicting results. DA seems to represent the main risk factor that leads to “reflection impulsivity”. Indeed, ICDs predominantly occurred subsequent to treatment initiation or dosage increase particularly related to the effects of the DA [36, 25]. However, an increase of impulsivity has been reported also after STN-DBS independently by dopaminergic medication status. DBS, indeed, has been described as “releasing the brake” of the STN, that lead to faster responses, particularly in a high-conflict situation, suggesting a diminished ability to hold initial response tendencies in check [42]. STN-DBS patients tend to prefer high-risk options and are (deliberately or not) overconfident [43]. One important characteristic of a decision-making network is inhibition of the prepotent response to each individual stimulus, thus avoiding rapid impulsive behavior that does not weigh up all options. STN-DBS seems to be associate with the inability to slow and integrate evidence before deciding, probably due to interference of stimulation with adjustments of decision thresholds. STN-DBS can raise action impulsivity, increasing response speed and lowering response accuracy (Ballanger et al., 2009). The speed–accuracy trade-off is a property of decision-making that can be controlled by cognitive processing.

Contrasting data suggest that STN-DBS significantly improves the proficiency of reactive inhibitory control [27, 41] and the suppression of irrelevant motor impulses. Selectively stimulating the dorsal as opposed to the ventral STN substructure is responsible for this effect [30]. Probably, the site of stimulation could moderate the improvement of ICD improvement. These suppositions underscore the importance of accurate electrode targeting, contact selection and device programming to reduce postoperative neuropsychiatric impairment. The ability to predict neuropsychiatric symptoms based on subthalamic data may permit anticipation and prevention of these occurrences, improving safety and tolerability [22]. STN-DBS coupled with a large reduction in dopaminergic medication has been shown to reduce pre-existing impulsive behavior, but the onset of new or worsening of existing ICDs in the post-operative period despite the reduction in dopaminergic medications has also been documented.

## Conclusion

The etiology and pathogenesis of treatment-induced impulsivity in PD remain unknown [44], though the altered activity of the mesolimbic dopamine system has been suggested to be responsible for the phenomenon, since dopaminergic neurons facilitate the adaptation of behavior according to reward or task demands. Similarly, it is well known that STN plays a part in the fronto-striato-thalamic-cortical loops mediating motor, cognitive, and emotional functions, thus suggesting that DBS may affect the behavior of PD patients, in addition to motor performance. However, literature data showed contrasting data about the role of DBS on impulsivity in PD patients. Several studies have stressed a direct correlation between STN-DBS and impulsive behavior, reporting the worsening or ex novo development of ICDs after surgery, other authors described a significant improvement of impulsivity after surgery. In addition, the studies reviewed involved only a limited number of participants, considering the high incidence of the disease and the sample heterogeneity, and the different tasks used did not permit a comparison between results. Future research should include the study of other factors, such as genetic predisposing, direct effect on the limbic part of STN, cognitive outcome or depression scores, and should conduct larger, prospective, controlled trials to better clarify how different subcomponents of impulsivity can be modulated both by dopaminergic drugs and STN-DBS.
